# Orbit period modulation for relative motion using continuous low thrust in the two-body and restricted three-body problems

**DOI:** 10.1007/s10569-017-9807-3

**Published:** 2018-01-18

**Authors:** C. S. Arnot, C. R. McInnes, R. J. McKay, M. Macdonald, J. Biggs

**Affiliations:** 1 Advanced Space Concepts Laboratory, Department of Mechanical and Aerospace Engineering, University of Strathclyde, Glasgow, UK; 2 School of Engineering, University of Glasgow, Glasgow, UK; 3 Department of Aerospace Science and Technology, Politecnico di Milano, Milan, Italy

**Keywords:** Non-Keplerian orbits, Continuous low thrust, Relative spacecraft motion, Orbit period modulation, Hill–Clohessy–Wiltshire equations, Restricted three-body problem

## Abstract

This paper presents rich new families of relative orbits for spacecraft formation flight generated through the application of continuous thrust with only minimal intervention into the dynamics of the problem. Such simplicity facilitates implementation for small, low-cost spacecraft with only position state feedback, and yet permits interesting and novel relative orbits in both two- and three-body systems with potential future applications in space-based interferometry, hyperspectral sensing, and on-orbit inspection. Position feedback is used to modify the natural frequencies of the linearised relative dynamics through direct manipulation of the system eigenvalues, producing new families of stable relative orbits. Specifically, in the Hill–Clohessy–Wiltshire frame, simple adaptations of the linearised dynamics are used to produce a circular relative orbit, frequency-modulated out-of-plane motion, and a novel doubly periodic cylindrical relative trajectory for the purposes of on-orbit inspection. Within the circular restricted three-body problem, a similar minimal approach with position feedback is used to generate new families of stable, frequency-modulated relative orbits in the vicinity of a Lagrange point, culminating in the derivation of the gain requirements for synchronisation of the in-plane and out-of-plane frequencies to yield a singly periodic tilted elliptical relative orbit with potential use as a Lunar far-side communications relay. The $\Delta v$ requirements for the cylindrical relative orbit and singly periodic Lagrange point orbit are analysed, and it is shown that these requirements are modest and feasible for existing low-thrust propulsion technology.

## Introduction

### Forced relative motion

After the advent of spacecraft rendezvous and docking, perhaps one of the earliest recognitions of the potential utility of spacecraft formation flight was in the form of a space-based interferometer, proposed by Sholomitsky et al. (1977), and then similarly by Labeyrie (1978). In the latter decades of the twentieth-century, the uses of multi-spacecraft missions were explored further, and indeed in recent years several missions using formation flight have flown, perhaps most prominently the ESA Cluster and NASA Magnetospheric Multiscale (MMS) missions.

The guidance and control of a wide variety of formation-flying concepts have been comprehensively surveyed in the previous decade, encompassing such applications as hyperspectral sensing and fractionated spacecraft (Scharf et al. 2003, 2004). Until very recently, such concepts generally assumed the use of conventional chemical propulsion for relative motion control. Low specific impulse and high thrust impose limitations on spacecraft formations, since the available $\Delta {v}$ is low and thrusters offer discrete impulses. Using such propulsion implies that the spacecraft must follow unforced ballistic trajectories between impulses. It has therefore been proposed that the continuous low thrust offered by modern electrostatic thrusters could add versatility to spacecraft formation flight (Austin et al. 1977). The thrust magnitudes required for formation keeping are generally small, and so several concepts for efficient electrostatic microthrusters have been developed (Cen and Xu 2010; Wirz et al. 2004). Alternatively, Coulomb forces for formation control have been investigated by several authors (e.g. Natarajan, and Schaub 2006; Schaub and Hussein 2010; King et al. 2003; Schaub et al. 2006).

In the restricted three-body problem, Dusek first proposed that artificial equilibrium points (AEPs) could be created in the vicinity of libration points using continuous thrust (Dusek 1966). Later, Cielaszyk and Wie presented a numerical method for halo orbit determination, where nonlinearities are considered persistent disturbance inputs (Cielaszyk and Wie 1996). More recently, Morimoto et al. investigated the thrust requirements to turn any arbitrary point in a restricted three-body system into an AEP, finding the regions of stable AEPs in small mass-ratio systems such as the Sun–Earth system which are accessible with small control accelerations (Morimoto et al. 2007). Other authors considered the use of active control to stabilise the motion of a spacecraft relative to a reference halo orbit in the Hill and restricted three-body problems, notably producing a circular relative trajectory with applications in formation flight for stellar interferometry (Scheeres et al. 2003; Hsiao and Scheeres 2005). Actively controlled formation flight in a two-body system has also been considered by several authors, such as Bando and Ichikawa (2013), where full-state feedback control has been used to force a spacecraft onto an arbitrary singly periodic reference orbit relative to an elliptical orbit.

Displacing the plane of an orbit using out-of-plane thrust was explored most prominently by Nock (1984), Yashko and Hastings (1996) and McInnes (1977). These displaced-plane orbits are often referred to as non-Keplerian orbits (NKOs), since the system barycentre is not in the orbit plane. The use of Solar sails to generate non-Keplerian geostationary orbits was considered by Baig and McInnes (2008) and Heiligers et al. (2012). Many authors have also established the existence, stability, and controllability conditions of such orbits (McInnes 1977, 1998; Scheeres 1999; Xu and Xu 2008; Bombardelli and Pelez 2011; Ceriotti et al. 2012; Aliasi et al. 2012), and recently McKay et al. performed a broad survey of NKOs and their utility (McKay et al. 2011). Recently, Wang et al. offered a new methodology for analysis of the formation flight of electric sails operating in NKOs, and subsequently presented a control framework for such sail formations (Wang et al. 2017a, b).

### Problem motivation and approach

In this paper, rich new families of relative trajectories for spacecraft formation flight are generated through the application of continuous thrust. In both the two-body Hill–Clohessy–Wiltshire frame and the circular restricted three-body problem, it is shown that simple position feedback can be used to modify the natural frequencies of the linearised dynamics through direct manipulation of the system eigenvalues and to thereby produce interesting and novel stable relative orbits. Whereas past authors have generally used a top-down engineering approach, designing active controllers with which to force a spacecraft onto a predetermined reference trajectory (e.g. Scheeres et al. 2003; Hsiao and Scheeres 2005; Bando and Ichikawa 2013), this paper instead seeks to generate rich new families of orbits with only position feedback and without a reference trajectory, thereby only minimally intervening into the dynamics of the problem. The assumption of the use of only position feedback instead of full-state feedback is justified by the goal of providing access to useful new trajectories for small, low-cost spacecraft equipped only with position sensing relative to a target spacecraft (e.g. Sansone et al. 2017). The use of only position feedback mitigates the difficulties in implementing accurate relative velocity sensing aboard such a spacecraft and also avoids the need for taking the time derivative of the position (a method which is inherently prone to noise errors), whilst still permitting attainment of relative orbits in both two- and three-body systems with potential future applications in space-based interferometry, hyperspectral sensing, and on-orbit inspection.

In 2010, NASA published a comprehensive study of on-orbit servicing, concluding that on-orbit servicing infrastructure was an essential and economical supporting step for future space missions (NASA GSFC 2010). It follows that on-orbit inspection is a necessary precursor to servicing, since it allows for advance detection and identification of points of failure aboard a satellite. In geostationary orbit, for example, many satellites could be inspected by a single small satellite or formations of small satellites in order to determine the need for servicing. Past authors have therefore proposed a number of free-flying strategies for on-orbit inspection (Woffinden 2004), and notably for space situational awareness (Erdner 2007) a 15 nanosatellite constellation tasked with inspecting the entire geostationary ring in less than a single year. However, the necessity of ballistic flight imposes limitations on an inspection mission—limitations which can be effectively addressed with the introduction of continuous low thrust.

One such major limitation of ballistic flight is that both the in-plane motion and decoupled out-of-plane motion of the spacecraft possess the same period, which is the reference orbit period. By making the system closed-loop and applying continuous low thrust proportional to the relative position of the spacecraft, it is possible to modify the natural period of the system. Due to the decoupling of the in-plane motion and out-of-plane motion, it is possible to have the case where the periods are distinct, and to cause the spacecraft to follow, for example, a helix of varying pitch around a target. This new and novel trajectory is a useful ability for on-orbit inspection as it allows for a detailed sweep of a target. Alternatively, for astronomy by a disaggregated spacecraft, a lens and formation of sensors could be made to rotate uniformly around a core spacecraft and thereby scan a large swathe of the sky.

The concept of using thrust proportional to relative position only to modify the natural periods of motion can also be usefully applied to the circular restricted three-body problem. Though the problem is normally nonlinear, the motion of a spacecraft in proximity to a Lagrange point can be linearised, and thrust proportional to position can be implemented. This can be used firstly to force the system to become linearly stable, and secondly to modify the natural frequencies of the motion. Further, the in-plane motion and out-of-plane motion can be coupled to yield a singly periodic orbit around the Lagrange point, which could be applied to provide a constantly visible communications relay for another spacecraft.

The structure of the paper is as follows. Section 2 builds on initial work (Arnot and McInnes 2015) concerning forced motion relative to a circular two-body reference orbit, and using a state-space approach systematically explores a number of different types of forced relative motion, with the final aim of generating a novel doubly periodic cylindrical relative orbit for on-orbit inspection of a target by a chase spacecraft. Section 3 concerns forced motion relative to a Lagrange point in the restricted three-body problem, using an approach similar to Sect. 2 and to recent authors such as Bando and Ichikawa (2013) to modify the natural frequencies of motion within regions of closed-loop stability, producing new and interesting types of multiply- and singly periodic relative trajectories. These novel relative orbits have wide ranging applications, such as for hyperspectral astronomy and the provision of constantly visible communications relays.

## Thrust augmented relative motion in the two-body rotating frame

This section comprises the systematic derivation and exploration of new families of forced relative orbits using linearised dynamics derived from the two-body problem. Using a state-space method, simple position feedback control will be used to manipulate the eigenvalues (and therefore the natural frequencies) of the system to produce interesting and novel new relative orbits. The primary aim of this section is to generate relative orbits with potential future applications in on-orbit inspection—an area where the advantages of modifying the frequencies of periodic motion are readily apparent.

**Fig. 1:**
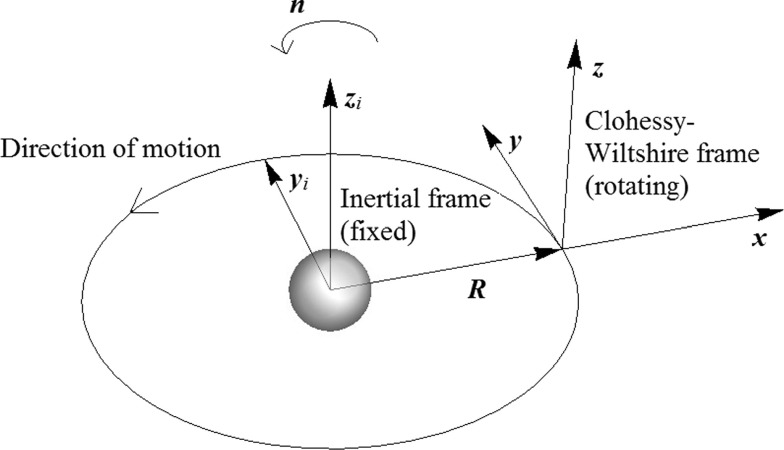
Rotating frame of reference

Figure 1 illustrates a rotating reference frame centred on a circular reference orbit about a point-mass central body. The motion of a chase spacecraft relative to a target on the reference orbit can be described by the linear Hill–Clohessy–Wiltshire (HCW) equations (Wiltshire and Clohessy 1960). With the target at the origin of the reference frame, we take the *x*-axis as following the radius vector from the central mass through the target, the *z*-axis following the orbital angular momentum vector, and the *y*-axis points in the along-track direction of the target’s motion around the central body. This reference frame forms the environment in which new relative orbits are generated throughout this section of the paper.

The well-known HCW equations, augmented with continuous thrust terms, are (Wiltshire and Clohessy 1960):

1a $$\begin{aligned} \ddot{x}= & {} 3n^2x+2n\dot{y}+u_x \end{aligned}$$

1b $$\begin{aligned} \ddot{y}= & {} -2n\dot{x}+u_y \end{aligned}$$

1c $$\begin{aligned} \ddot{z}= & {} -n^2z+u_z, \end{aligned}$$

where $u_x$, $u_y$, and $u_z$ are the thrust-induced acceleration terms, and *n* is the angular velocity of the rotating frame. This is in turn given by

2 $$\begin{aligned} n=\sqrt{\mu /R^3} \end{aligned}$$

in which $\mu$ is the gravitational parameter and *R* is the inertial-frame orbit radius.

To apply controlling thrust terms to the HCW equations, the motion from Eq. (1a–1c) is first converted to the state-space form

3 $$\begin{aligned} \dot{\varvec{x}}=\varvec{A}\varvec{x}+\varvec{B}\varvec{u}, \end{aligned}$$

where $$\varvec{x}=[x~~y~~z~~\dot{x}~~\dot{y}~~\dot{z}]^T$$, and

4 $$\begin{aligned}&\displaystyle \varvec{A}=\begin{bmatrix} 0&\quad 0&\quad 0&\quad 1&\quad 0&\quad 0\\ 0&\quad 0&\quad 0&\quad 0&\quad 1&\quad 0\\ 0&\quad 0&\quad 0&\quad 0&\quad 0&\quad 1\\ 3n^2&\quad 0&\quad 0&\quad 0&\quad 2n&\quad 0\\ 0&\quad 0&\quad 0&\quad -2n&\quad 0&\quad 0\\ 0&\quad 0&\quad -n^2&\quad 0&\quad 0&\quad 0 \end{bmatrix}&\end{aligned}$$

5 $$\begin{aligned}&\displaystyle \varvec{B}=\begin{bmatrix} 0&\quad 0&\quad 0 \\ 0&\quad 0&\quad 0 \\ 0&\quad 0&\quad 0 \\ 1&\quad 0&\quad 0\\ 0&\quad 1&\quad 0 \\ 0&\quad 0&\quad 1 \end{bmatrix}.&\end{aligned}$$

An extremely simple strategy is proposed whereby the thrust-induced acceleration is proportional to the displacement in the radial, along-track, and out-of-plane axes only. The acceleration-law is defined by

6 $$\begin{aligned} \varvec{u}=-\varvec{K}\varvec{x}. \end{aligned}$$

The feedback gain matrix $\varvec{K}$ is given by

7 $$\begin{aligned} \varvec{K}=\begin{bmatrix} K_{11}&\quad 0&\quad 0&\quad 0&\quad 0&\quad 0\\ 0&\quad K_{22}&\quad 0&\quad 0&\quad 0&\quad 0\\ 0&\quad 0&\quad K_{33}&\quad 0&\quad 0&\quad 0 \end{bmatrix}. \end{aligned}$$

The upper bound of the input acceleration can be easily defined therefore in terms of the maximum displacement in each axis, as

8 $$\begin{aligned} \varvec{u}_{\mathrm{max}}=\begin{bmatrix} |{u_{x \mathrm{max}}}|\\ |{u_{y \mathrm{max}}}|\\ |{u_{z \mathrm{max}}}|\end{bmatrix}=\begin{bmatrix} K_{11} x_{\mathrm{max}}\\ K_{22} y_{\mathrm{max}}\\ K_{33} z_{\mathrm{max}} \end{bmatrix}. \end{aligned}$$

The control acceleration can therefore be bounded through the appropriate selection of the maximum displacement in each axis, which is in turn generally determined from the initial conditions.

Now, set $\varvec{A_c}=\varvec{A}-\varvec{B}\varvec{K}$, which has eigenvalues $\varvec{\lambda }$ and corresponding eigenvectors $\varvec{V}$. The general eigenvalues are found to be

9 $$\begin{aligned} \varvec{\lambda }=\begin{bmatrix} -\frac{\sqrt{-K_{11}-K_{22}-n^2-\sqrt{(K_{11}-K_{22})^2+2(K_{11}+7K_{22})n^2+n^4}}}{\sqrt{2}} \\ \frac{\sqrt{-K_{11}-K_{22}-n^2-\sqrt{(K_{11}-K_{22})^2+2(K_{11}+7K_{22})n^2+n^4}}}{\sqrt{2}} \\ -\frac{\sqrt{-K_{11}-K_{22}-n^2+\sqrt{(K_{11}-K_{22})^2+2(K_{11}+7K_{22})n^2+n^4}}}{\sqrt{2}} \\ \frac{\sqrt{-K_{11}-K_{22}-n^2+\sqrt{(K_{11}-K_{22})^2+2(K_{11}+7K_{22})n^2+n^4}}}{\sqrt{2}} \\ -\sqrt{-K_{33}-n^2} \\ \sqrt{-K_{33}-n^2} \end{bmatrix}. \end{aligned}$$

Since the eigenvalues represent the natural frequencies of the system, modifying $\varvec{K}$ therefore directly modifies these frequencies. Feedback gains $K_{11}$ and $K_{22}$ both affect the first two conjugate pairs of eigenvalues corresponding to the in-plane motion, and $K_{33}$ only affects a single decoupled pair of eigenvalues, corresponding to the *z*-axis motion. This key idea of modifying the natural frequencies of the system through feedback is used to produce interesting and novel relative trajectories in both the two-body and subsequent three-body sections of this paper.

**Fig. 2:**
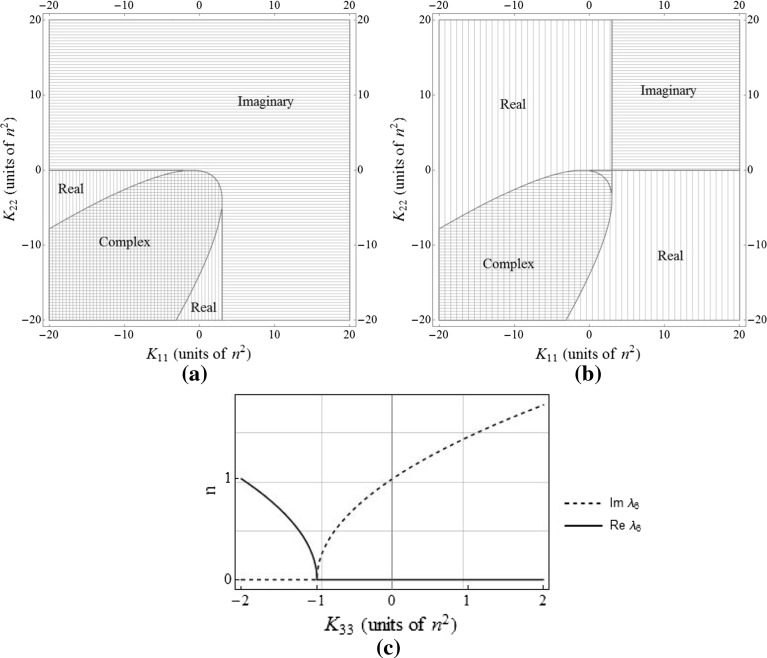
Regions for which (**a**) $\lambda _2$, (**b**) $\lambda _4$, and (**c**) $\lambda _6$ are purely imaginary, purely real, and complex

Stable oscillatory behaviour occurs when the eigenvalues are imaginary, so it is useful to find the corresponding range of gains for this behaviour. Considering the second, fourth, and sixth elements of Eq. (9), $\lambda _2$, $\lambda _4$, and $\lambda _6$, since these eigenvalues each form one half of a conjugate pair, plots indicating the regions in which these eigenvalues are real, complex, and imaginary are given in Fig. 2. Unstable regions are found where the real parts of the eigenvalues are greater than zero. The eigenvalue $\lambda _6$ is considered separately since it is only affected by a single gain, $K_{33}$.

The state transition matrix, $\varvec{\varPhi }$, which can be used to find the general solution to the system, is given by

10 $$\begin{aligned} \varvec{\varPhi }=\varvec{W}(t)\varvec{W}^{-1}(0). \end{aligned}$$

The fundamental solution to the system, $\varvec{W}(t)$, can be found using

11 $$\begin{aligned} \varvec{W}(t)=\varvec{V}\begin{bmatrix} e^{\lambda _1t}&\quad 0&\quad \cdots&\quad 0\\ 0&\quad e^{\lambda _2t}&\quad \cdots&\quad 0\\ \vdots&\quad&\quad \ddots&\quad \vdots \\ 0&\quad \cdots&\quad&\quad e^{\lambda _6t} \end{bmatrix}. \end{aligned}$$

Note that, in the case where the eigenvalues are complex, it is necessary to take the real and imaginary parts of the complex solution separately to find the real fundamental solution to the system, $\varvec{W_r}(t)$. The general solution to the system, including control inputs, can then be found using

12 $$\begin{aligned} \varvec{x}(t)=\varvec{W_r}(t)\varvec{W_r}^{-1}(0)\varvec{x}(0)+\varvec{W_r}(t)\int _{t_0}^{t}\varvec{W_r}^{-1}(\tau )\varvec{B}\varvec{u}\,\mathrm{d}\tau . \end{aligned}$$

In the case that $K_{11}=K_{22}=K_{33}=0$, the time domain solution to the system is equal to the well-known closed-form solutions to the HCW equations, shown in Eq. (13):

13 $$\begin{aligned}&\begin{bmatrix} x(t)\\ y(t)\\ z(t)\\ \dot{x}(t)\\ \dot{y}(t)\\ \dot{z}(t) \end{bmatrix}=\begin{bmatrix} 4-3\cos nt&\quad 0&\quad 0&\quad \frac{1}{n}\sin nt&\quad \frac{2}{n}(1-\cos nt)&\quad 0\\ 6(\sin nt-nt)&\quad 1&\quad 0&\quad -\frac{2}{n}(1-\cos nt)&\quad \frac{1}{n}(4\sin nt-3nt)&\quad 0\\ 0&\quad 0&\quad \cos nt&\quad 0&\quad 0&\quad \frac{1}{n}\sin nt\\ 3n\sin nt&\quad 0&\quad 0&\quad \cos nt&\quad 2 \sin nt&\quad 0\\ -6(1-\cos nt)&\quad 0&\quad 0&\quad -2 \sin nt&\quad 4\cos nt-3&\quad 0\\ 0&\quad 0&\quad -n\sin nt&\quad 0&\quad 0&\quad \cos nt \end{bmatrix}\begin{bmatrix} x_0\\ y_0\\ z_0\\ \dot{x}_0\\ \dot{y}_0\\ \dot{z}_0 \end{bmatrix}.\nonumber \\ \end{aligned}$$

Since the aim of this work is to modify the natural frequencies of the system and thereby generate novel trajectories, it is necessary that at least one of the gains is nonzero, and so the solution in Eq. (13) cannot be used in its entirety. However, part of this solution will be used in certain $\Delta v$ calculations later in this section.

### **Artificial equilibria and a simple circular relative orbit**

A simple yet interesting case to demonstrate the eigenvalue-based approach is that of the generation of artificial static equilibria in the rotating frame using continuous thrust, since, if nonzero initial velocity is *not* assumed, it is useful to characterise the type and frequency of motion followed by the spacecraft. Such equilibria in the rotating frame are equivalent to type III non-Keplerian orbits when viewed from an inertial frame (McInnes 1977).

Consider the state vector $\varvec{x}=[x,y,z,\dot{x},\dot{y},\dot{z}]^{T}$ in which $\dot{x}$, $\dot{y}$, and $\dot{z}$ must be zero for a static displacement. Since $\varvec{u}=-\varvec{Kx}$, the system state equation is $\dot{\varvec{x}}=\varvec{A}\varvec{x}+\varvec{B}(-\varvec{Kx})$. With zero initial velocity ($\dot{x}_0=\dot{y}_0=\dot{z}_0=0$) the system state is held constant by selecting the feedback gains as

14 $$\begin{aligned} \varvec{K}=\begin{bmatrix} 3n^2&\quad 0&\quad 0&\quad 0&\quad 0&\quad 0\\ 0&\quad 0&\quad 0&\quad 0&\quad 0&\quad 0\\ 0&\quad 0&\quad -n^2&\quad 0&\quad 0&\quad 0 \end{bmatrix}. \end{aligned}$$

With reference to Fig. 2, the motion should be stable, with all nonzero eigenvalues being imaginary. Indeed, the eigenvalues of the system are found to be

15 $$\begin{aligned} \varvec{\lambda }=\begin{bmatrix} -2in \\ 2in \\ 0 \\ 0\\ 0\\ 0 \end{bmatrix}. \end{aligned}$$

**Fig. 3:**
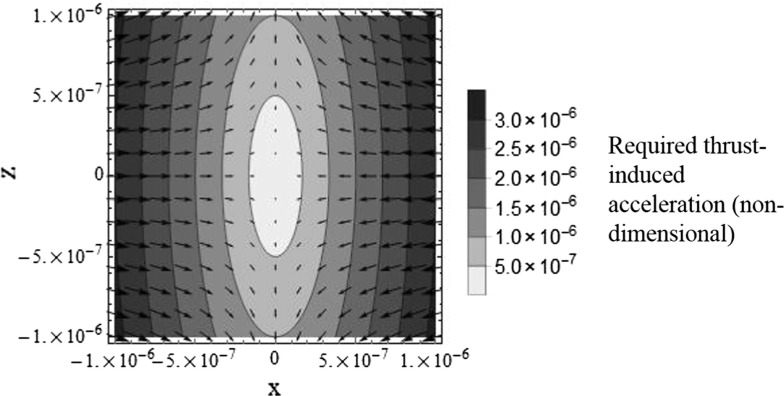
Required thrust vector field for static formations in the *x*-*z* plane, where the position is normalised by the reference orbit radius, and the acceleration is normalised by the reference orbit radius and orbit period

If the initial velocity is zero, there are no oscillations and the spacecraft remains fixed at its initial position. However, with nonzero initial velocity, since the coefficient of both $\lambda _1$ and $\lambda _2$ is 2, the forced natural frequency of the motion in the *x*–*y* plane is twice the unforced natural frequency. Since $K_{22}=0$, for static formations in the rotating frame, the along-track position is arbitrary as it does not affect the required thrust. For a thrust-induced acceleration vector $\varvec{u}$ of fixed magnitude, a required thrust vector field for static equilibrium positions in the *x*–*z* plane can be generated, as shown in Fig. 3 where all values are normalised by the reference orbit radius. All subsequent plots of relative orbits in the two-body section are also normalised in this way.

**Fig. 4:**
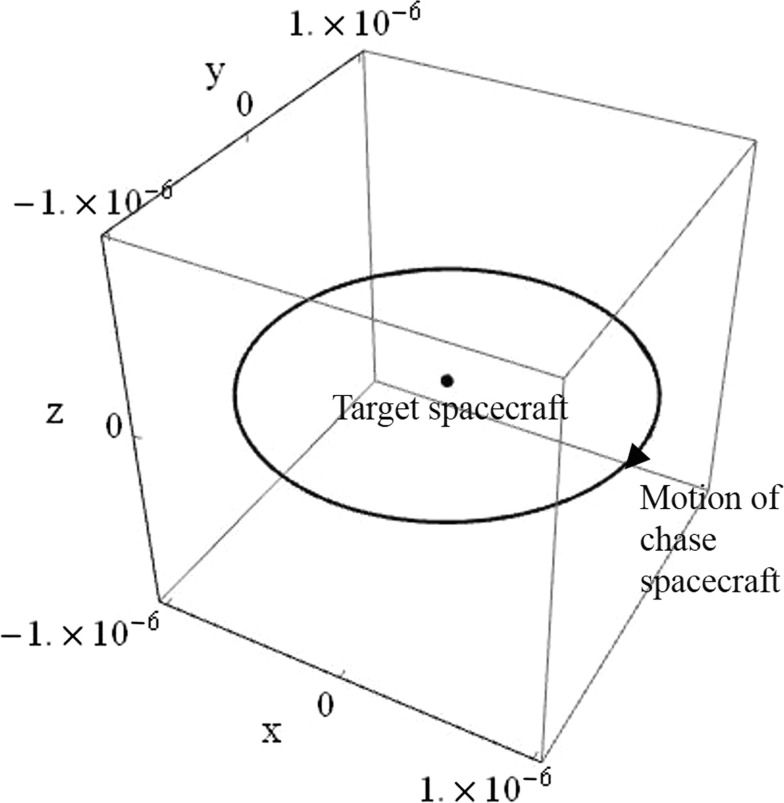
Non-dimensional in-plane circular relative orbit achieved with single axis thrust

Interestingly, the in-plane motion of the spacecraft can be made circular in this case simply by selecting the appropriate initial velocity in the *x*- and *y*-directions. Recalling the well-known condition for bounded motion in the HCW equations, $\dot{y}_0=-\,2nx_0$, it is necessary only to add the condition $\dot{x}_0=2ny_0$, and with the feedback gains of Eq. (14) the result is a circular trajectory in the *x*–*y* plane. As already indicated, the relative orbit period is half of the reference orbit period. Furthermore, in this case the gain $K_{33}$ is arbitrary because the *z*-axis motion is decoupled and the circular trajectory exists only in the *x*–*y* plane, and so the circular relative orbit is achieved using thrust in only the radial direction. An example of this type of relative motion is plotted in Fig. 4.

In the case of zero initial velocity, i.e. for a static formation, the $\Delta V$ required to maintain the formation is simple to calculate. Since with zero initial velocity $u_x$ is constant, and assuming independent body-mounted thrusters on each axis, it is possible to use

16 $$\begin{aligned} \Delta v_x=3n^2x\tau , \end{aligned}$$

where $\tau$ is the duration for which the formation is maintained. Similarly, since $u_z$ is also constant, we can use

17 $$\begin{aligned} \Delta v_z=n^2z\tau . \end{aligned}$$

Using an example of a geostationary target, with Eqs. (16) and (17), the $\Delta V$ accumulated for a chase spacecraft positioned for one sidereal day in a 100 m *z*-axis statically displaced NKO is 0.046 m s$^{-1}$, and for a 100 m *x*-axis displacement is 0.138 m s$^{-1}$. Assuming the use of electrostatic ion thrusters with a specific impulse of 3000 s, for a nanosatellite with initial mass of 10 kg, this amounts to a propellant expenditure of only $1.56\times 10^{-5}$ and $4.69\times 10^{-5}$ kg, respectively.

### **Modulation of the out-of-plane period**

Having considered the in-plane behaviour of the system under the feedback gains of Eq. (14), the out-of-plane motion is now considered. When $K_{33}=-\,n^2$ with some nonzero $z_0$, the chase spacecraft is fixed in a displaced non-Keplerian orbit whose plane does not contain the two-body centre of mass. Oppositely, when $K_{33}=0$, we have the ballistic case, and the spacecraft oscillates along the *z*-axis with a period equal to the reference orbit period. A *z*-displaced static formation can therefore be considered a periodic relative orbit with infinite out-of-plane period. As noted by Arnot and McInnes (2015), it is possible to modify the period of the periodic *z*-axis motion by making the *z*-axis thrust proportional to displacement. The period of the motion along the *z*-axis is now modified by changing the out-of-plane thrust component.

To begin, it is necessary to substitute $K_{33}=-\,n^2$ with $K_{33}=-\,\psi ^2$, so that the eigenvalue corresponding to the out-of-plane motion, $\lambda _6$ in Eq. (9), becomes

18 $$\begin{aligned} \lambda _6=\sqrt{\psi ^2-n^2}. \end{aligned}$$

It follows that

19 $$\begin{aligned} \varvec{A_c}=\begin{bmatrix} 0&\quad 0&\quad 0&\quad 1&\quad 0&\quad 0\\ 0&\quad 0&\quad 0&\quad 0&\quad 1&\quad 0\\ 0&\quad 0&\quad 0&\quad 0&\quad 0&\quad 1\\ -K_{11}+3n^2&\quad 0&\quad 0&\quad 0&\quad 2n&\quad 0\\ 0&\quad -K_{22}&\quad 0&\quad -2n&\quad 0&\quad 0\\ 0&\quad 0&\quad \psi ^2-n^2&\quad 0&\quad 0&\quad 0 \end{bmatrix}. \end{aligned}$$

The augmented angular frequency, $\varOmega$, is defined by

20 $$\begin{aligned} \varOmega ^2=(n^2-\psi ^2) \end{aligned}$$

and by

21 $$\begin{aligned} \varOmega =\frac{n}{k} \end{aligned}$$

in which *k* represents the number of reference orbit periods in which the thrust augmented relative motion completes a single out-of-plane cycle. Therefore, *k* can be considered the augmented period coefficient, so that the period of the *z*-axis motion is $T_z=kT$. Through use of out-of-plane thrust, the period of the *z*-axis motion can now be freely chosen.

Substituting Eq. (21) into (20), it is possible to rearrange for $\psi$ such that

22 $$\begin{aligned} \psi =n\sqrt{1-\Big (\frac{1}{k^2}\Big )}. \end{aligned}$$

It can then be shown that the equation of out-of-plane motion is given by

23 $$\begin{aligned} \ddot{z}=-\,\frac{n^2z}{k^2}, \end{aligned}$$

which is a harmonic oscillator whose natural frequency can now be selected through the coefficient of the out-of-plane thrust law. The maximum input acceleration for this type of relative orbit is given simply by $|u_{z \mathrm{max}}|=\psi ^2z_0$, assuming that the initial velocity is zero.

**Fig. 5:**
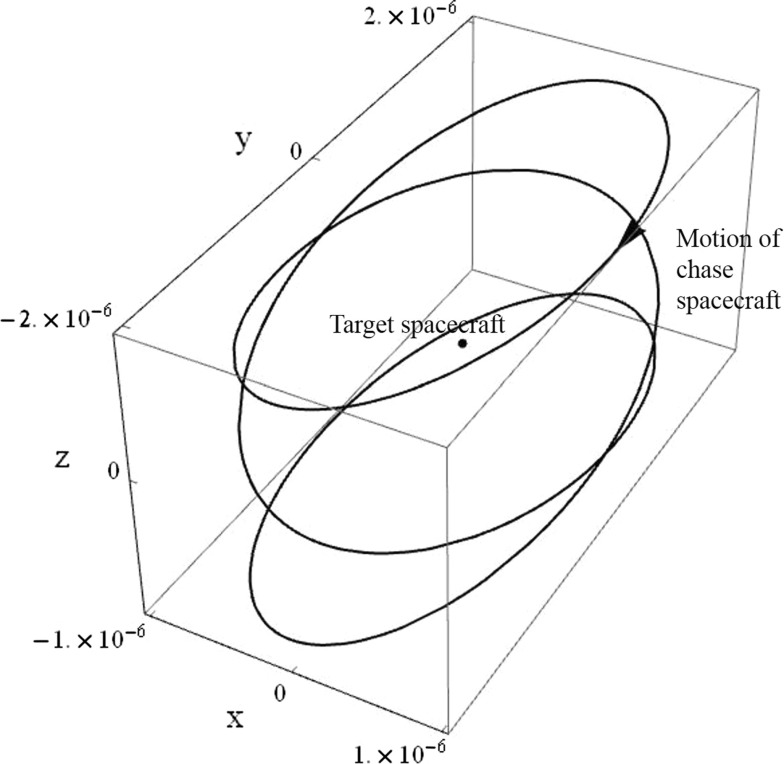
Relative orbit with forced out-of-plane period of 3*T*

An example of thrust augmented *z*-axis motion is shown in Fig. 5, where $k=3$, $x_0=z_0=0.5$, and $\dot{y}_0=-2nx_0$, simulated for three reference orbit periods.

Clearly, when $k=1$, the thrust-induced acceleration in the *z*-direction is zero, corresponding to ballistic motion. However, when $k<1$, the thrust is nonzero and in the opposite direction to the case $k>1$. In addition, the frequency of oscillation in the *z*-direction is greater than the unforced frequency. When $k\rightarrow \infty$, the expression for the thrust acceleration simplifies to $u_z=n^2z$, so $K_{33}=-\,n^2$, and the *z*-axis displacement becomes fixed: that is, the trajectory is equivalent to static equilibria in the rotating frame.

To calculate the $\Delta v$ required to maintain this type of continuously forced orbit, we must first consider the third row of Eq. (13) which describes the unforced out-of-plane position of the chase spacecraft. Since $\dot{z}_0=0$ and the frequency of the out-of-plane motion is now defined by $\varOmega$, the out-of-plane displacement simplifies to

24 $$\begin{aligned} z(t)=z_0\cos \varOmega t. \end{aligned}$$

It follows that the thrust command, $u_z$, becomes

25 $$\begin{aligned} u_z=\psi ^2z_0\cos \varOmega t. \end{aligned}$$

To find the $\Delta v$ accumulated over multiple orbit periods, the magnitude of the acceleration must be considered since the direction of the out-of-plane thrust changes direction at every plane crossing. This requires description as a piecewise function, such that

26 $$\begin{aligned} \vert u_z \vert ={\left\{ \begin{array}{ll} \psi ^2z_0\cos \varOmega t~\qquad if~\cos \varOmega t\ge 0 \\ -\psi ^2z_0\cos \varOmega t~\quad if~\cos \varOmega t<0 \end{array}\right. }. \end{aligned}$$

The integral then takes the form (Arnot and McInnes 2015)

27 $$\begin{aligned} \Delta v=\psi ^2 z_0\left[ \eta \int _{t=0}^{t=T_z}\vert \cos \varOmega t\vert \mathrm{d}t+\int _{t=\eta T_z}^{t=\varepsilon +\eta T_z}\vert \cos \varOmega t\vert \mathrm{d}t\right] , \end{aligned}$$

where $\eta$ is the integer number of complete *z*-axis motion periods which have elapsed and $\varepsilon$ is the additional time over the integer number of periods. Thus, the expression for accumulated $\Delta v$ becomes

28 $$\begin{aligned} \Delta v=\psi ^2z_0\left[ \frac{4\eta }{\varOmega }+\int _{t=\eta T_z}^{t=\varepsilon +T_z} \vert \cos \varOmega t\vert \mathrm{d}t \right] . \end{aligned}$$

Equation (28) can be integrated for all positive integer values of $\eta$, and for $0\le \varepsilon <T_z$.

### The cylindrical relative orbit

Now that a means to modulate the frequency of the out-of-plane motion is possessed, an interesting and novel application can be envisaged: on-orbit inspection of a target by a chase spacecraft using continuous thrust to modify its relative orbit period. The concept of on-orbit inspection has been explored by other authors; however, the use of continuous low thrust has generally not been considered in this context. Using continuous thrust, a chase spacecraft on an inspection mission can actively force its relative motion to enable operationally advantageous new Keplerian and non-Keplerian inspection trajectories. Perhaps the most commercially viable example is that of a small spacecraft tasked to inspect multiple satellites on the geostationary ring, and so previous work has considered the use of a thrust augmented relative orbit in which the chase spacecraft tracks the Sun vector around a target in geostationary orbit (Arnot and McInnes 2015). Such a trajectory makes use of constant-angle sunlight to facilitate visual inspection. Here the more general case of a cylindrical relative orbit is considered, which makes use of thrust augmented in-plane motion and out-of-plane motion to produce an orbit with two distinct modified periods.

A simple cylindrical relative orbit can be achieved by using the circular in-plane orbit already described (using $K_{11}=3n^2$ and $K_{22}=0$) combined with out-of-plane orbit period modulation ($K_{33}=-\psi ^2$). Selecting an appropriate out-of-plane period, the result is that the chase spacecraft performs a helical sweep around the target as it oscillates between $z_0$ and $-z_0$ with an in-plane period of 0.5*T*. However, although it is possible to freely select the out-of-plane period, this type of trajectory has a fixed in-plane period. A circular relative orbit whose period and orientation can also be freely selected would be of greater operational advantage. Bando and Ichikawa (2013) showed that circular and elliptical relative orbits of arbitrary frequency were achievable using active control; however, a useful analytical description of such a family of orbits has apparently not been presented in the literature and is derived parametrically as follows.

To produce a circular trajectory about the target spacecraft, consider first a three-dimensional circle described parametrically by the vector $\varvec{\alpha }=[\alpha _1,\alpha _2,\alpha _3]^T$, which is collinear with the circle’s transformed *x*-axis (transformed from the *x*-axis of the rotating frame and fixed with respect to the circle), and the vector $\varvec{\beta }=[\beta _1,\beta _2,\beta _3]^T$, which is collinear with the transformed *y*-axis of the circle. Both $\varvec{\alpha }$ and $\varvec{\beta }$ are unit vectors. The vector describing the position of the circle’s centre in the rotating frame is $\varvec{c}=[c_1,c_2,c_3]^T$, $\varvec{r}$ is the radius vector of the circle, and $\theta$ is the angle between the radius vector and the *x*-axis as measured in the anticlockwise direction about the circle’s central axis. It is taken that $\theta =-\gamma nt$ (negative because the motion of the chase spacecraft in the *x*-*y*-plane is clockwise), where $\gamma$ is the ratio of the target spacecraft’s Keplerian orbit period to that of the circular relative orbit in the rotating frame (for instance, if $\gamma =1$, the period of the circular motion is equal to that of the Keplerian orbit of the target spacecraft), and where *n* and *t* have their usual meaning. The inclusion of $\gamma$ permits the modification of the period of the relative orbit, so that

29 $$\begin{aligned} \varvec{r}=\varvec{c}+r\cos (\theta )\varvec{\alpha }+r\sin (\theta )\varvec{\beta }. \end{aligned}$$

The first and second derivatives of Eq. (29) are found to be

30 $$\begin{aligned}&\displaystyle \varvec{\dot{r}}=r\gamma n\sin (\theta )\varvec{\alpha }-r\gamma n\cos (\theta )\varvec{\beta }&\end{aligned}$$

31 $$\begin{aligned}&\displaystyle \varvec{\ddot{r}}=-r(\gamma n)^2\cos (\theta )\varvec{\alpha }-r(\gamma n)^2\sin (\theta )\varvec{\beta }.&\end{aligned}$$

It follows that, in this case, $\varvec{\dot{x}}=[\varvec{\dot{r}}, \varvec{\ddot{r}}]^T$. Then, referring to Eq. (3), we recall the acceleration input $\varvec{u}=[u_x, u_y, u_z]^T$. Thus, it can be shown that

32 $$\begin{aligned} \varvec{\dot{x}}=\begin{bmatrix} \dot{x}\\ \dot{y}\\ \dot{z}\\ 3n^2 x+2n\dot{y}+u_x\\ -2n\dot{x}+u_y\\ -n^2 z+u_z \end{bmatrix}=\begin{bmatrix} r\gamma n\sin (\theta )\alpha _1-r\gamma n\cos (\theta )\beta _1\\ r\gamma n\sin (\theta )\alpha _2-r\gamma n\cos (\theta )\beta _2\\ r\gamma n\sin (\theta )\alpha _3-r\gamma n\cos (\theta )\beta _4\\ -r(\gamma n)^2\cos (\theta )\alpha _1-r(\gamma n)^2\sin (\theta )\beta _1\\ -r(\gamma n)^2\cos (\theta )\alpha _2-r(\gamma n)^2\sin (\theta )\beta _2\\ -r(\gamma n)^2\cos (\theta )\alpha _3-r(\gamma n)^2\sin (\theta )\beta _3 \end{bmatrix}. \end{aligned}$$

The input acceleration $\varvec{u}$ can then be obtained as

33 $$\begin{aligned} \varvec{u}=\begin{bmatrix} n^2(-r(\alpha _1(\gamma ^2+3)-2\beta _2\gamma )\cos \gamma nt+r(2\alpha _2\gamma +\beta _1(\gamma ^2+3))\sin \gamma nt-3c_1\\ -n^2r\gamma ((2\alpha _1-\beta _2\gamma )\sin \gamma nt+(\alpha _2\gamma +2\beta _1)\cos \gamma nt)\\ n^2(-\alpha _3r(\gamma ^2-1)\cos \gamma nt+\beta _3r(\gamma ^2-1)\sin \gamma nt+c_3 \end{bmatrix}. \end{aligned}$$

Using $u_x$ from Eq. (33), it can be shown that the single axis thrust case described earlier is a special case of the general thrust equations. Since the special case is a circle in the *x*-*y* plane and centred at the origin, $\varvec{\alpha }=[1,0,0]^T$, $\varvec{\beta }=[0,1,0]^T$, and $\varvec{c}=[0,0,0]^T$, $\varvec{u}$ simplifies to

34 $$\begin{aligned} \varvec{u}=\begin{bmatrix} -n^2 r(\gamma ^2-2\gamma +3)\cos \gamma nt\\ -n^2 r \gamma (2-\gamma )\sin \gamma nt\\ 0 \end{bmatrix}, \end{aligned}$$

where $r\cos \gamma nt\equiv x$. Therefore, in order for the first row of Eq. (34) to be equivalent to $u_x=-\,3n^2x$, it is clear that $\gamma =2$. Interestingly, we note that with $\gamma =2$, it follows that $u_y=0$ and as required, $u_x=-\,3n^2r\cos 2nt\equiv -3n^2x$, which is equivalent to the earlier state feedback case where $K_{11}=3n^2$. This provides a particularly simple steering law with a useful application. The value of $\gamma$ also correctly indicates that the period of the in-plane motion is 0.5*T*. From Eq. (34), it is deduced that the maximum input acceleration in each axis is given by $|u_{x\mathrm{max}}|=-n^2 r(\gamma ^2-2\gamma +3)$ and $|u_{y\mathrm{max}}|=-n^2 r \gamma (2-\gamma )$ for the *x*-axis and *y*-axis, respectively.

**Fig. 6:**
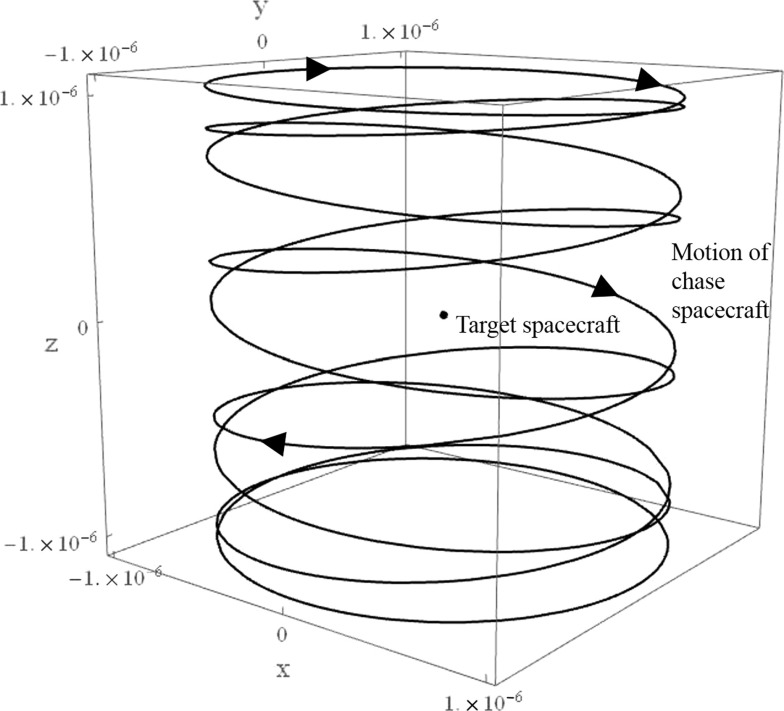
Cylindrical relative orbit with $\gamma =1$ and $k=9$

The general case thrust commands of Eq. (33) can be used to produce a circular relative orbit with arbitrary dimension, orientation, and frequency. However, for the cylindrical relative orbit, the circle is only required in the *x*-*y* plane, and so the vectors $\varvec{\alpha }$ and $\varvec{\beta }$ are collinear with the rotating frame *x*- and *y*-axes in this case. The two unit vectors $\varvec{\alpha }$ and $\varvec{\beta }$ are $[1,0,0]^T$ and $[0,1,0]^T$, respectively. The circle’s centre is $c=[0,0,z]$. Now, using the in-plane thrust components defined in Eq. (34) and the out-of-plane gain $K_{33}=-\psi ^2$, the natural frequencies of the circular in-plane motion and oscillatory out-of-plane motion can be freely modified. As before, the *z*-axis position varies sinusoidally between the maximum and minimum displacement. An example cylindrical relative orbit achieved using this approach is displayed in Fig. 6, with non-dimensional axes.

The $\Delta \varvec{v}$ required to maintain the cylindrical orbit is found by integrating the three thrust acceleration components and summing the result, assuming independent body-mounted thrusters. Taking the *x* and *y* components from Eq. (34), the moduli are

35a $$\begin{aligned} \vert u_x \vert= & {} {\left\{ \begin{array}{ll} \vert -n^2r(\gamma ^2-2\gamma +3)\vert \cos \gamma nt~{ if}~\cos \gamma nt\ge 0 \\ -n^2r(\gamma ^2-2\gamma +3)\cos \gamma nt~{ if}~\cos \gamma nt<0 \end{array}\right. } \end{aligned}$$

35b $$\begin{aligned} \vert u_y \vert= & {} {\left\{ \begin{array}{ll} \vert -n^2r\gamma (2-\gamma )\vert \sin \gamma nt~{ if}~\sin \gamma nt\ge 0 \\ -n^2r\gamma (2-\gamma )\sin \gamma nt~{ if}~\sin \gamma nt<0. \end{array}\right. } \end{aligned}$$

The integrals then become (Arnot and McInnes 2015)

36a $$\begin{aligned} \Delta v_x= & {} \vert -n^2r(\gamma ^2-2\gamma +3)\vert \left[ q\int _{t=0}^{t=T_{xy}}\vert \cos \gamma nt\vert \mathrm{d}t+\int _{t=qT_{xy}}^{t=\upsilon +qT_{xy}}\vert \cos \gamma nt\vert \mathrm{d}t\right] \nonumber \\\end{aligned}$$

36b $$\begin{aligned} \Delta v_y= & {} \vert -n^2r\gamma (2-\gamma )\vert \left[ q\int _{t=0}^{t=T_{xy}}\vert \sin \gamma nt\vert \mathrm{d}t+\int _{t=qT_{xy}}^{t=\upsilon +qT_{xy}}\vert \sin \gamma nt\vert \mathrm{d}t\right] , \end{aligned}$$

where $\Delta v_x$ and $\Delta v_y$ are the components of $\Delta \varvec{v}$ in the *x*- and *y*-directions, respectively, $T_{xy}$ is the period of the *x*-*y* planar motion, *q* is the integer number of *x*-*y* planar motion periods which have elapsed, and $\upsilon$ is the additional time over the integer number of *x*-*y* motion periods. Integrating, the expressions for $\Delta \varvec{v}$ become

37a $$\begin{aligned} \Delta v_x= & {} \vert -n^2r(\gamma ^2-2\gamma +3)\vert \left[ \frac{4q}{\gamma n}+\int _{t=qT_{xy}}^{t=\upsilon +qT_{xy}}\vert \cos \gamma nt\vert \mathrm{d}t\right] \end{aligned}$$

37b $$\begin{aligned} \Delta v_y= & {} \vert -n^2r\gamma (2-\gamma )\vert \left[ \frac{4q}{\gamma n}+\int _{t=qT_{xy}}^{t=\upsilon +qT_{xy}}\vert \sin \gamma nt\vert \mathrm{d}t\right] . \end{aligned}$$

The *z*-axis component of $\Delta \varvec{v}$ is as given in Eq. (28). Equations (37a) and (37b) can be integrated for all positive integer values of *q*, and for $0\le \upsilon <T_{xy}$. Using the Sun vector tracking orbit (which orbits a geostationary target with an in-plane period of one Solar day and out-of-plane period of one year) as an example of this type of orbit (Arnot and McInnes 2015), with a 100-m in-plane radius, 43.3 m maximum out-of-plane displacement, and independent axis-aligned thrusters, the total $\Delta v$ for a full year of operation would be 36.7 m s$^{-1}$. This would amount to 0.0125 kg of propellant for a 10 kg nanosatellite equipped with electrostatic thrusters with specific impulse of 3000 s.

**Fig. 7:**
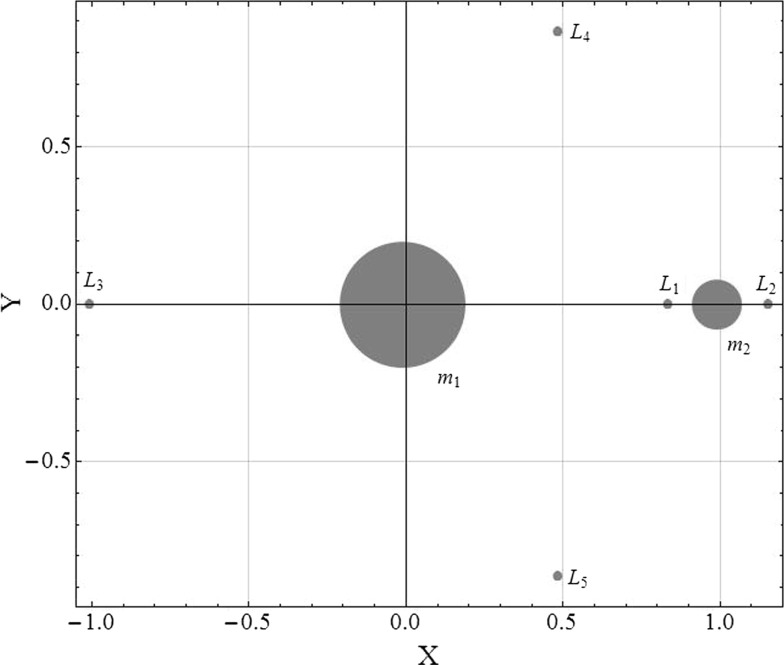
Schematic of the circular restricted three-body problem with $\rho =0.01213$ (equivalent to the Earth–Moon system)

## Thrust augmented orbits at Lagrange points

Thus far, this paper has considered only relative motion around a circular reference orbit using linearised dynamics derived from the two-body problem. However, a similar approach can be used for the generation of thrust augmented relative orbits in the vicinity of a Lagrange point, in the circular restricted three-body problem (CRTBP), using linearised dynamics for spacecraft relative motion. Whereas the previous section considered the generation of orbits with potential applications for on-orbit inspection, this section aims to derive the feedback gains necessary to synchronise the in-plane and out-of-plane frequencies to produce a stable, three-dimensional, singly periodic orbit with potential utility as an Earth–Moon $L_2$ communications relay with constant visibility from the Earth. A special case of this kind of orbit, controlled using solar radiation pressure, was investigated by Tanaka and Kawaguchi (2016) and can further be considered an extension of Farquhar’s original concept for a Lunar far-side communications relay (Farquhar 1968).

In Fig. 7, the layout of the CRTBP rotating frame is given. Within this frame, a spacecraft’s position vector is normalised using the system’s circular orbit radius, and the time is normalised by the orbit period. The orbit period is taken to be $2\pi$ and the normalised time is *t*. The usual origin of the system is at the barycentre of the two primary masses, the axis *X* is aligned with the vector connecting the two primary masses ($m_1$ and $m_2$) and *Y* is perpendicular to it such that the *X*-*Y* plane is the Earth–Moon plane. However, since the aim is to examine the motion of a low-thrust spacecraft relative to a Lagrange point, it is useful to place the origin of the system at the Lagrange point in question, $L_i$. Using this new origin, *x* is aligned with the *X*-axis, *y* is aligned with the *Y*-axis, and *z* completes the right-hand coordinate system. The linearised equations of relative motion in the vicinity of the Lagrange point are given by Farquhar (1968)

38a $$\begin{aligned} \ddot{x}= & {} 2{\dot{y}}+(2\sigma _i+1)x \end{aligned}$$

38b $$\begin{aligned} \ddot{y}= & {} (1-\sigma _i)y-2\dot{x} \end{aligned}$$

38c $$\begin{aligned} \ddot{z}= & {} -\sigma _iz. \end{aligned}$$

It is taken that

39 $$\begin{aligned} \sigma _i=\frac{\rho }{|l_i(\rho )-1+\rho |^3}+\frac{(1-\rho )}{|l_i(\rho )+\rho |^3} \end{aligned}$$

in which $l_i(\rho )$ is the distance of the collinear Lagrange point from the system barycentre. The mass ratio $\rho$ is given by

40 $$\begin{aligned} \rho =\frac{m_2}{m_1+m_2}. \end{aligned}$$

The distance of the Lagrange point from the system barycentre can be found by solving the quintic expression resulting from

41 $$\begin{aligned} l_i(\rho )=\frac{1-\rho }{r_1^3}(l_i(\rho )+\rho )+\frac{\rho }{r_2^3}(l_i(\rho )-1+\rho ). \end{aligned}$$

The general state-space form has the same construction as Eq. (3), where $\varvec{x}=[x~y~z~\dot{x}~\dot{y}~\dot{z}]^T$, $\varvec{u}$ has the same form as Eq. (6), and $\varvec{K}$ is the same as Eq. (7). The system matrix $\varvec{A}$ has the new form

42 $$\begin{aligned} \varvec{A}=\begin{bmatrix} 0&0&0&1&0&0\\ 0&0&0&0&1&0\\ 0&0&0&0&0&1\\ 2\sigma _i+1&0&0&0&2&0\\ 0&1-\sigma _i&0&-2&0&0\\ 0&0&-\sigma _i&0&0&0 \end{bmatrix} \end{aligned}$$

and $\varvec{B}$ is the same as Eq. (5). Using the relation $\varvec{A_c}=\varvec{A}-\varvec{BK}$ which has eigenvalues $\varvec{\lambda }$ and eigenvectors $\varvec{V}$, the general eigenvalues of the closed-loop system are found to be

43 $$\begin{aligned} \varvec{\lambda }=\begin{bmatrix} -\frac{\sqrt{-2-K_{11}-K_{22}+\sigma _i-\sqrt{K_{11}^2+8(K_{22}-\sigma _i)-2K_{11}(-4+K_{22}+3\sigma _i)+(K_{22}+3\sigma _i)^2}}}{\sqrt{2}} \\ \frac{\sqrt{-2-K_{11}-K_{22}+\sigma _i-\sqrt{K_{11}^2+8(K_{22}-\sigma _i)-2K_{11}(-4+K_{22}+3\sigma _i)+(K_{22}+3\sigma _i)^2}}}{\sqrt{2}} \\ -\frac{\sqrt{-2-K_{11}-K_{22}+\sigma _i+\sqrt{K_{11}^2+8(K_{22}-\sigma _i)-2K_{11}(-4+K_{22}+3\sigma _i)+(K_{22}+3\sigma _i)^2}}}{\sqrt{2}} \\ \frac{\sqrt{-2-K_{11}-K_{22}+\sigma _i+\sqrt{K_{11}^2+8(K_{22}-\sigma _i)-2K_{11}(-4+K_{22}+3\sigma _i)+(K_{22}+3\sigma _i)^2}}}{\sqrt{2}} \\ -\sqrt{-K_{33}-\sigma _i} \\ \sqrt{-K_{33}-\sigma _i} \end{bmatrix}. \end{aligned}$$

**Fig. 8:**
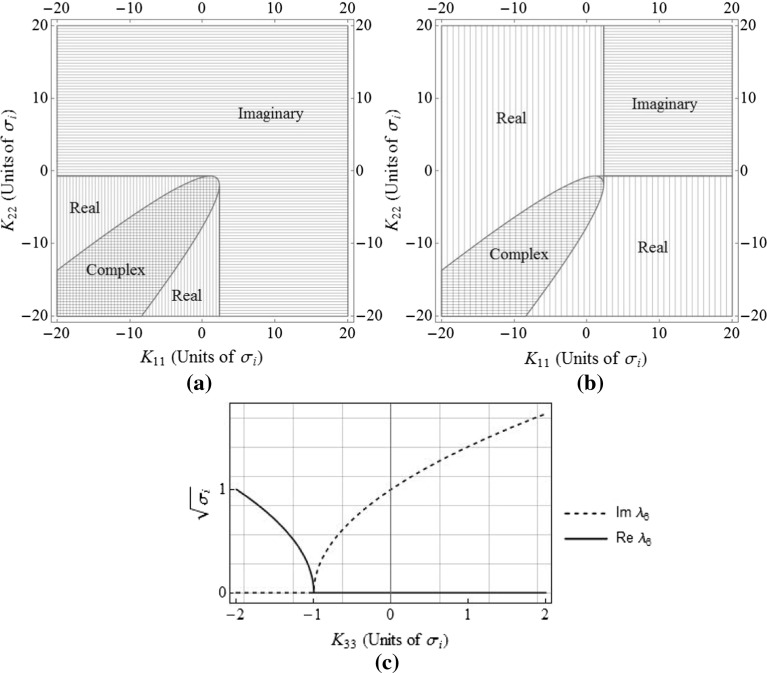
Regions for which (**a**) $\lambda _2$, (**b**) $\lambda _4$, and (**c**) $\lambda _6$ are purely imaginary, purely real, and complex, for Earth–Moon $L_2$ point $(\sigma _2 = 3.19097)$

Thus, in similar fashion to the linearised two-body problem, it is possible to modify the natural frequencies of the in-plane motion and out-of-plane motion independently by modifying $\varvec{K}$. For the Earth–Moon $L_2$ point ($\rho =0.01213$, $\sigma _2=3.19097$) the regions where the eigenvalues are purely imaginary, real, or complex are shown in Fig. 8. These regions are similar to those of the two-body system, however unlike the two-body case the region boundaries are dependent on $\sigma _i$ and so the stable region changes at different Lagrange points and with different $\rho$.

With reference to Fig. 8, it is worth noting that, unlike within the two-body HCW system, the in-plane motion around the Earth–Moon $L_2$ is naturally unstable. With $K_{11}$ and $K_{22}$ both at zero, $\lambda _4$ is positive and real. Therefore, in order to design useful, stable, oscillatory relative trajectories around the Lagrange point, it is necessary to apply feedback gains such that the eigenvalues become purely imaginary. For the Earth–Moon $L_2$ point, the relative in-plane motion will be stable and oscillatory when gains of approximately $K_{11}>2.28\sigma _2$ and $K_{22}>-0.65\sigma _2$ are selected.

**Fig. 9:**
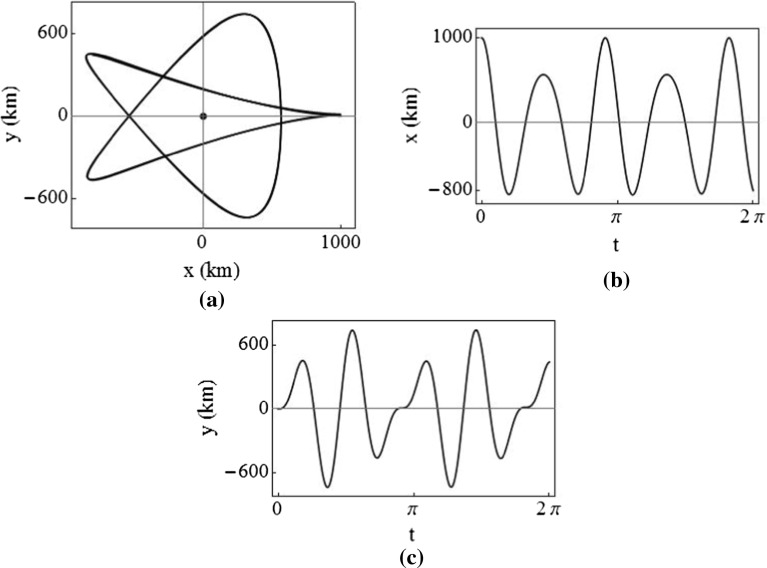
In-plane trajectory for the doubly periodic case, using $K_{11}=K_{22}=10\sigma _2$, for Earth–Moon $L_2$ ($\sigma _2=3.19097$)

For arbitrary initial conditions, with $K_{11}$ and $K_{22}$ in the stable region, interesting doubly periodic trajectories are produced. An example of this is shown in Fig. 9, where $K_{11}=K_{22}=10 \sigma _2$ and the initial velocity is zero. Since the *z*-axis motion is decoupled, it is not shown here.

Let $\varvec{p}_j+i\varvec{q}_j$ be the eigenvector corresponding to eigenvalue $\lambda _j$, and consider only the two in-plane eigenvalues $\lambda _2$ and $\lambda _4$ (since the out-of-plane motion is decoupled) such that $j=(2, 4)$. Recalling that the eigenvalue $\lambda _j$ is equivalent to the natural frequency $\omega _j$, it can be shown that the three-dimensional periodic solution is found from Baig (2009)

44 $$\begin{aligned} \begin{bmatrix} x\\ y\\ \dot{x}\\ \dot{y}\\ \end{bmatrix}= & {} \cos (\omega _2t)[A\varvec{p}_2+B\varvec{q}_2]+\sin (\omega _2t)[B\varvec{p}_2-A\varvec{q}_2] \nonumber \\&+\cos (\omega _4t)[C\varvec{p}_4+D\varvec{q}_4]+\sin (\omega _4t)[D\varvec{p}_4-C\varvec{q}_4]. \end{aligned}$$

From this, by setting $t=0$, the initial conditions are

45 $$\begin{aligned} \begin{bmatrix} x_0\\ y_0\\ \dot{x}_0\\ \dot{y}_0\\ \end{bmatrix}= \begin{bmatrix} -2\omega _2B-2\omega _4D\\ A(h+\omega _2^2)+C(h+\omega _4^2)\\ 2\omega _2^2A+2\omega _4^2C\\ \omega _2(h+\omega _2^2)B+\omega _4(h+\omega _4^2)D \end{bmatrix}. \end{aligned}$$

The constants *A*, *B*, *C*, and *D* are then

46 $$\begin{aligned} \begin{bmatrix} A\\ B\\ C\\ D\\ \end{bmatrix}= \begin{bmatrix} -\frac{-h\dot{x}_0-\dot{x}_0\omega _4^2+2y_0\omega _4^2}{2h(\omega _2^2-\omega _4^2)}\\ -\frac{-2\dot{y}_0-hx_0-x_0\omega _4^2}{2\omega _2(\omega _2^2-\omega _4^2)}\\ -\frac{-h\dot{x}_0-\dot{x}_0\omega _2^2+2y_0\omega _2^2}{2h(\omega _4^2-\omega _2^2)}\\ -\frac{-2\dot{y}_0-hx_0-x_0\omega _2^2}{2\omega _4(\omega _4^2-\omega _2^2)} \end{bmatrix}. \end{aligned}$$

The initial conditions required for $C=0$ and $D=0$ are then found to be

47 $$\begin{aligned} \begin{bmatrix} \dot{x}_0\\ \dot{y}_0\\ \end{bmatrix}= \begin{bmatrix} \frac{2y_0\omega _2^2}{h+\omega _2^2}\\ \frac{-x_0(h+\omega _2^2)}{2} \end{bmatrix}. \end{aligned}$$

**Fig. 10:**
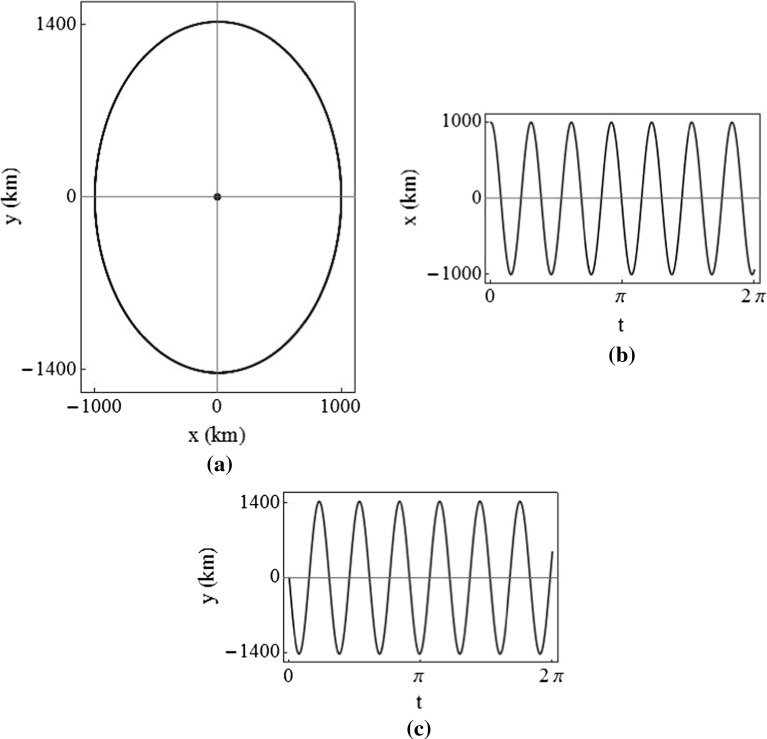
Periodic in-plane trajectory, using $K_{11}=K_{22}=10\sigma _2$

So a periodic solution with the same form as the ballistic periodic solution (Baig 2009) and dependence on a single natural frequency $\omega _2$ is found as

48a $$\begin{aligned} x(t)= & {} -A_x\cos (\omega _{2}t+\phi ) \end{aligned}$$

48b $$\begin{aligned} y(t)= & {} kA_x\sin (\omega _{2}t+\phi ) \end{aligned}$$

in which $k=\frac{(\omega _{2}^2+h)}{2\omega _{2}}$, $A_x$ is the amplitude of the *x*-axis motion, and $\phi$ is the phase angle. Both $K_{11}$ and $K_{22}$ can now be varied to change the natural frequency and *y*-axis amplitude of the elliptical relative orbit around the Lagrange point. The out-of-plane motion, which is always periodic, has the solution

49 $$\begin{aligned} z(t)=A_z\sin (\omega _{6}t+\phi _z), \end{aligned}$$

where $A_z$ is the *z*-axis amplitude, $\phi _z$ is the phase angle, and $\omega _6$ is the out-of-plane natural frequency which can be modified by changing $K_{33}$. The periodic in-plane motion generated by these initial conditions is shown in Fig. 10, where $K_{11}=K_{22}=10\sigma _2$ and the initial velocity is as in Eq. (47).

**Fig. 11:**
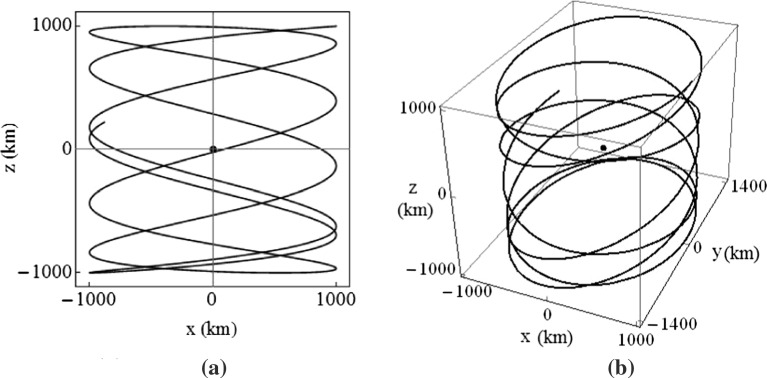
Thrust augmented Lissajous trajectory about Earth–Moon $L_2$ point, using $K_{11}=K_{22}=10\sigma _2$

It can then be shown that 3-D periodic orbits are achieved when $\omega _2/\omega _6$ is a rational number, and quasi-periodic Lissajous trajectories are attained when $\omega _2/\omega _6$ is irrational. An example of a thrust augmented Lissajous trajectory is plotted in Fig. 11, where $K_{11}=K_{22}=10\sigma _2$ and $K_{33}=0$.

For $\omega _2=\omega _6$, it is necessary that

50 $$\begin{aligned} K_{33}=\frac{2{+}K_{11}{+}K_{22}{-}3\sigma _i{+}\sqrt{K_{11}^2{+}8(K_{22}{-}\sigma _i){-}2K_{11}(-4{+}K_{22}+3\sigma _i)+(K_{22}+3\sigma _i)^2}}{2}. \nonumber \\ \end{aligned}$$

**Fig. 12:**
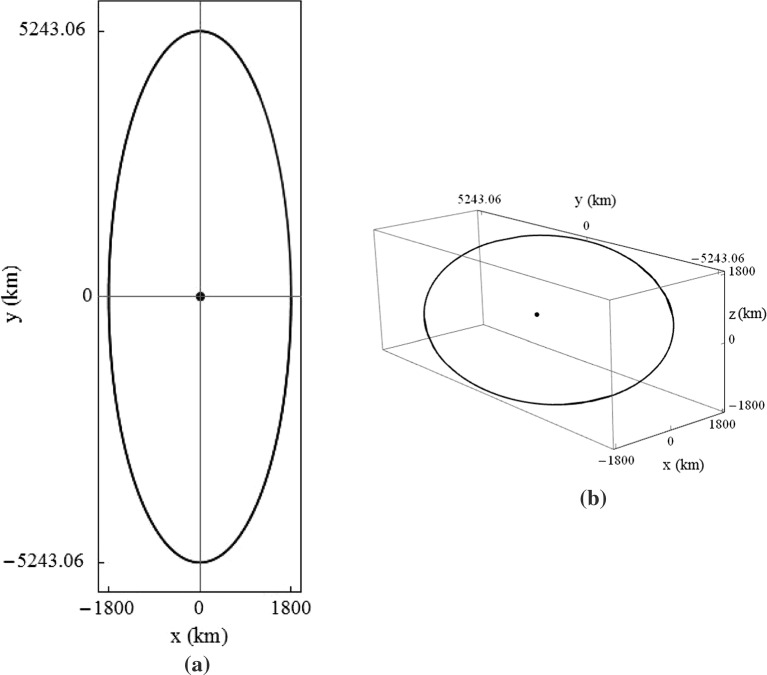
Trajectory around Earth–Moon $L_2$ point when $\omega _{xy}=\omega _z$, using $K_{11}=K_{22}=0$

This produces a tilted periodic orbit with a potential application in providing an $L_2$ communications relay for the far side of the Moon with respect to the Earth, as an extension of the concept first proposed by Farquhar (1968): instead of out-of-plane phase-jump control impulses, continuous low thrust with gain governed by Eq. (50) is used to prevent occultation of the spacecraft behind the Moon. Constant line of sight with Earth is achieved by ensuring that $A_x$ and $A_z$ are greater than the radius of the Moon’s relative umbra. The in-plane and 3-D trajectories of a spacecraft on such an orbit around the Earth–Moon $L_2$ are shown in Fig. 12, for $A_x=A_z=1800$ km. For this example orbit, with zero thrust in the in-plane directions, the peak thrust-induced acceleration in the *z*-axis is $3.56\,\upmu \hbox {m s}^{-2}$. The required $\Delta v_z$ for one year of operation is $74.4\hbox { m s}^{-1}$, corresponding to a 0.025 kg propellant expenditure for a spacecraft with initial mass of 10 kg, equipped with electrostatic thrusters of $I_{sp}=3000$ s.

## Conclusions

Continuous low-thrust propulsion can augment the capabilities of spacecraft formations by allowing relative flight on forced Keplerian and non-Keplerian trajectories. Simple control strategies using only position feedback can be applied to generate rich new families of stable relative orbits in both the two-body and restricted three-body problems, whilst requiring only minimal intervention into the dynamics of the problem, to permit implementation aboard small, low-cost spacecraft. One main contribution of this paper is the parametric derivation of a forced circular orbit of arbitrary dimension, orientation, and period relative to a target on a circular two-body orbit. A second contribution is the derivation of thrust commands for out-of-plane period modulation, leading to the analytical description of a cylindrical relative orbit in the two-body rotating frame, with potential future applications in on-orbit inspection. A third contribution is the derivation of gain requirements for the synchronisation of in-plane and out-of-plane periods of a relative orbit around a Lagrange point in the restricted three-body problem, such that a stable, singly periodic 3-D trajectory can be accessed for the purposes of providing a constantly visible Earth–Moon $L_2$ communications relay. Further, the propellant requirements for each new orbit type are relatively small and achievable when the use of modern electrostatic thrusters is assumed.

## References

[CR1] Aliasi G, Mengali G, Quarta AA (2012). Artificial equilibrium points for a generalized sail in the elliptic restricted three-body problem. Celest. Mech. Dyn. Astron..

[CR2] Arnot, C.S., McInnes, C.R.: Low thrust augmented spacecraft formation-flying for on-orbit inspection. In: 66th International Astronautical Congress, IAC-15-C1.8.1.x28589 (2015)

[CR3] Austin RE, Dod RE, Terwilliger CH (1977). The ubiquitous solar electric propulsion stage. Acta Astronaut..

[CR4] Baig, S.: Non-Keplerian orbits for low-thrust propulsion, Ph.D. thesis, Department of Mechanical Engineering, University of Strathclyde (2009)

[CR5] Baig S, McInnes CR (2008). Artificial three-body equilibria for hybrid low-thrust propulsion. J. Guid. Control Dyn..

[CR6] Bando, M., Ichikawa, A.: Formation flying near the libration points in the elliptic restricted three-body problem. In: 5th International Conference on Spacecraft Formation Flying Missions and Trajectories, Munich, Germany (2013)

[CR7] Bando M, Ichikawa A (2013). Active formation flying along an elliptic orbit. J. Guid. Control Dyn..

[CR8] Bombardelli C, Pelez J (2011). On the stability of artificial equilibrium points in the circular restricted three-body problem. Celest. Mech. Dyn. Astron..

[CR9] Cen JW, Xu JL (2010). Performance evaluation and flow visualization of a MEMS based vaporizing liquid micro-thruster. Acta Astronaut..

[CR10] Ceriotti M, McInnes CR (2012). Natural and sail-displaced doubly-symmetric Lagrange point orbits for polar coverage. Celest. Mech. Dyn. Astron..

[CR11] Cielaszyk D, Wie B (1996). New approach to halo orbit determination and control. J. Guid. Control Dyn..

[CR12] Dusek HM (1966). Motion in the vicinity of libration points of a generalized restricted three-body model. Prog. Astronaut. Aeronaut..

[CR13] Erdner, M.T.: Smaller satellite operations near geostationary orbit, M.Sc. thesis, Naval Postgraduate School, Monterey, CA (2007)

[CR14] Farquhar, R.W.: The Control and Use of Libration-Point Satellites, Ph.D. thesis, Department of Aeronautics and Astronautics, Stanford University, Stanford, California (1968)

[CR15] Heiligers J, McInnes CR, Biggs JD, Ceriotti M (2012). Displaced geostationary orbits using hybrid low-thrust propulsion. Acta Astron..

[CR16] Hsiao FY, Scheeres DJ (2005). Design of spacecraft formation orbits relative to a stabilized trajectory. J. Guid. Control Dyn..

[CR17] King LB, Parker GG, Deshmukh S, Chong J-H (2003). Study of interspacecraft Coulomb forces and implications for formation flying. J. Propuls. Power.

[CR18] Labeyrie A (1978). Stellar interferometry methods. Ann. Rev. Astron. Astrophys..

[CR19] McInnes CR (1997). The existence and stability of families of displaced two-body orbits. Celest. Mech. Dyn. Astron..

[CR20] McInnes CR (1998). Dynamics, stability, and control of displaced non-Keplerian orbits. J. Guid. Control Dyn..

[CR21] McKay RJ, Macdonald M, Biggs J, McInnes CR (2011). Survey of highly non-Keplerian orbits with low-thrust propulsion. J. Guid. Control Dyn..

[CR22] Morimoto MY, Yamakawa H, Uesugi K (2007). Artificial equilibrium points in the low-thrust restricted three-body problem. J. Guid. Control Dyn..

[CR23] NASA GSFC.: On-Orbit Satellite Servicing Study Project Report, NASA Project Report NP-2010-08-162-GSFC, Greenbelt, MD (2010)

[CR24] Natarajan A, Schaub H (2006). Linear dynamics and stability analysis of a two-craft Coulomb tether formation. J. Guid. Control Dyn..

[CR25] Nock KT (1984). Rendezvous with Saturns Rings, Planetary Rings: 1st International Meeting.

[CR26] Sansone F, Branz F, Francesconi A (2017). A Relative Navigation Sensor for Cubesats Based on Retro-Reflective Markers, 2017 IEEE International Workshop on Metrology for AeroSpace.

[CR27] Scharf, D.P., Hadaegh, F.Y., Ploen, S.R.: A survey of spacecraft formation flying guidance and control (part I): guidance. In: Proceedings of the 2003 American Control Conference, vol. 2, pp. 1733–1739. Denver, Colorado (2003). 10.1109/ACC.2003.1239845

[CR28] Scharf, D.P., Hadaegh, F.Y., Ploen, S.R.: A survey of spacecraft formation flying guidance and control (part II): control. In: Proceedings of the 2004 American Control Conference, vol. 4, pp. 2976–2985. Boston, Massachusetts (2004)

[CR29] Schaub H, Hussein II (2010). Stability and reconfiguration analysis of a circularly spinning two-craft Coulomb tether. IEEE Trans. Aerosp. Electron. Syst..

[CR30] Schaub H, Hall CD, Berryman J (2006). Necessary conditions for circularly-restricted static Coulomb formations. J. Astronaut. Sci.

[CR31] Scheeres DJ (1999). Stability of hovering orbits around small bodies. Adv. Astron. Sci..

[CR32] Scheeres DJ, Hsiao F-Y, Vinh NX (2003). Stabilizing motion relative to an unstable orbit: applications to spacecraft formation flight. J. Guid. Control Dyn..

[CR33] Sholomitsky, G.B., Prilutsky, O.F., Rodin, V.G.: Infra-red space interferometer. In: 28th International Astronautical Federation Congress, Prague, Czechoslovakia, IAF-77-68 (1977)

[CR34] Tanaka K, Kawaguchi J (2016). Small-amplitude periodic orbit around Sun–Earth L1/L2 controlled by solar radiation pressure. Trans. Jpn. Soc. Aeronaut. Space Sci..

[CR35] Wang, W., Mengali, G., Quarta, A.A., Yuan, J.: Formation flying for electric sails in displaced orbits. Part I: geometrical analysis. Adv. Space Res. (2017a). 10.1016/j.asr.2017.05.015

[CR36] Wang, W., Mengali, G., Quarta, A.A., Yuan, J.: Formation flying for electric sails in displaced orbits. Part II: distributed coordinated control. Adv. Space Res. (2017b). 10.1016/j.asr.2017.06.017

[CR37] Wiltshire RS, Clohessy WH (1960). Terminal guidance system for satellite rendezvous. J. Aerospace Sci..

[CR38] Wirz, R., Gale, M., Mueller, J., Marrese, C.: Miniature ion thruster for precision formation flying. In: 40th AIAA/ASME/SAE/ASEE Joint Propulsion Conference and Exhibit, Fort Lauderdale, Florida, AIAA. pp. 2004–4115 (2004). 10.2514/6.2004-4115

[CR39] Woffinden, D.C.: On-orbit satellite inspection, M.Sc. thesis, Massachusetts Institute of Technology, Cambridge, MA (2004)

[CR40] Xu M, Xu S (2008). Nonlinear dynamical analysis for displaced orbits above a planet. Celest. Mech. Dyn. Astron..

[CR41] Yashko, G.J., Hastings, D.E.: Analysis of thruster requirements and capabilities for local satellite clusters. In: 10th AIAA/USU Conference on Small Satellites, AIAA, Logan, Utah (1996)

